# Poisoning by Atropia Applied to the Conjunctiva

**Published:** 1853-12

**Authors:** 


*Poisoning by Atropia applied to the Conjunctiva.*—The Gazette des Hopitaux gives an account of a case of poisoning by atropia, which is remarkable for the small quantity employed, and the place of application—viz., the healthy conjunctiva. A patient in the Hospital Saint Antoine was laboring under double cataract, complicated with adhesions of the iris to the lens. In order to ascertain exactly the condition of the eyes, three or four drops of a solution, containing about one grain of atropine to two ounces of water acidulated with acetic acid, were instilled into each eye. Half an hour afterwards the patient suffered from vertigo, and complained of disagreeable, unusual sensations; three quarters of an hour later he manifested all the symptoms of poisoning by belladonna,—redness and animation of the face—pupils, though irregular, enormously dilated, incessant hallucinations, seizing the bedclothes, and grasping at objects in the air. He could not raise himself from his bed, nor advance a step without being supported, as his limbs trembled and gave way at every effort. Pulse full, and beating 120. These symptoms gradually passed off, but the patient did not recover his normal condition for three or four days afterwards. Being interrogated upon the delirium and hallucinations of the preceding days, he had only a vague recollection of what had passed.—*Dublin Med. Press.*

